# Elevated soluble cellular adhesion molecules are associated with increased mortality in a prospective cohort of renal transplant recipients

**DOI:** 10.1186/1471-2369-12-23

**Published:** 2011-05-22

**Authors:** Grainne M Connolly, Ronan Cunningham, Peter T McNamee, Ian S Young, Alexander P Maxwell

**Affiliations:** 1 Department of Clinical Biochemistry, Royal Victoria Hospital, Belfast, Northern Ireland; 2 Regional Nephrology Unit, Belfast City Hospital, Belfast, Northern Ireland

## Abstract

**Background:**

Increased plasma levels of cellular adhesion molecules (CAMs) have been shown to be predictors of all cause mortality in individuals with chronic renal failure [1,2] and patients with end-stage renal disease receiving haemodialysis [3]. In renal transplant recipients the predictive value of CAMs has not been well characterised. The aim of this study was to assess the relationship between CAMs and all-cause mortality during prospective follow-up of a renal transplant cohort.

**Methods:**

A total of 378 renal transplant recipients were recruited between June 2000 and December 2002. Soluble vascular CAM-1 (VCAM) and soluble intercellular CAM-1 (ICAM) were measured at baseline and prospective follow-up data was collected at a median of 2441 days after enrolment.

**Results:**

In univariate survival analysis the renal transplant recipients with a VCAM or ICAM concentration in the lowest third were significantly more likely to have survived at follow-up (p < 0.001 and p = 0.009 respectively). In multivariate survival analysis VCAM and ICAM remained significant independent predictors of mortality following adjustment for traditional cardiovascular risk factors, hsCRP and estimated GFR (p = 0.030 and p = 0.037 respectively).

**Conclusions:**

The results of this prospective study are the first to show that the CAMs, ICAM and particularly VCAM, are significant independent predictors of mortality in patients with a renal transplant.

## Background

Patients with chronic kidney disease (CKD) have an increased prevalence of atherosclerosis as compared to the general population. However traditional cardiovascular risk factors do not adequately account for the increased burden of vascular disease in persons with CKD [4]. Since inflammation has been implicated in all stages of the development of atherosclerosis [5], attention has been focused on molecules involved in inflammatory pathways and the utility of such biomarkers to identify individuals at increased risk of cardiovascular events.

Cell-to-cell interactions are critical at every phase in the development of the atherosclerotic plaque and cellular adhesion molecules are essential mediators in this process, playing a central role in the recruitment of inflammatory cells to the site of atheroma development [6].

Following activation, cellular adhesion molecules are shed from the surface of endothelial cells [7] and can be measured in plasma [8]. Soluble cellular adhesion molecules (CAMs) therefore represent promising biomarkers that may reflect underlying endothelial activation and vascular inflammation [9].

Many factors have been shown to alter expression of cellular adhesion molecules. These include hypertension [10] immunosuppressive therapy [11], autoimmune disease [11-13] and cell mediated allograft rejection [14,15].

However increased plasma levels of CAMs have been identified in persons with atherosclerotic disease [6] and elevated concentrations of CAMs are significant predictors of future death from cardiovascular causes among patients with documented coronary artery disease [16].

Elevated concentrations of CAMs are found in persons with chronic kidney disease and reduced glomerular filtration rates [17,18]. In pre-dialysis patients, soluble intercellular CAM-1 (ICAM) [1,2] and soluble vascular CAM-1 (VCAM) [2] have been shown to be independent predictors of mortality. Similarly, CAMs have been reported to be predictors of all cause mortality in patients with end-stage renal disease receiving haemodialysis [3].

However, the predictive value of CAMs has not been well defined in renal transplant recipients. We hypothesised that CAMs would predict risk of death in patients with a renal transplant. The aim of this study therefore was to assess the relationship between the CAMs, ICAM and VCAM, and all-cause mortality during the prospective follow-up of a renal transplant cohort.

## Methods

Between June 2000 and December 2002, 378 renal transplant recipients were enrolled into this observational prospective study. Participants were recruited from the renal transplant clinics at Belfast City Hospital and Antrim Area Hospital in Northern Ireland, United Kingdom.

The study was approved by the Research Ethics Committee Queen's University Belfast and fully informed written consent was obtained from each participant prior to enrolment. Patients were eligible for entry if they had a functioning renal transplant present. No formal exclusion criteria were imposed. However, patients who were clinically unwell or had signs of sepsis at initial assessment were deferred until a subsequent clinic re-assessment.

All the renal transplant patients recruited to this study were greater than 2 months post-renal transplant and 94% were recruited more than 12 months after transplant surgery. All participants had stable graft function and were on standard immunosuppression regimens.

The 378 renal transplant recipients enrolled in this study represented 71.7% of all patients with a functioning renal transplant in Northern Ireland at the end of 2002 and 98% of all renal transplant recipients attending the transplant clinics at Belfast City Hospital and Antrim Area Hospital. The patients not enrolled either did not consent to participate in the study or, more commonly, were attending renal transplant clinics at other geographically distant hospitals in the Northern Ireland region.

At enrolment, with the assistance of a research nurse, each participant completed a cardiovascular risk assessment questionnaire. This recorded drug history, the presence of traditional cardiovascular risk factors (age, gender, diabetes and smoking history) and history of vascular disease. Prior vascular disease was defined as history of stroke, myocardial infarction, coronary artery bypass grafting, angioplasty, amputation for peripheral vascular disease or angiographic evidence of atherosclerotic vascular disease.

Each participant also had a measurement of blood pressure. This was recorded as the average of the last three blood pressure measurements (measured using Disytest sphygmomanometer Welch-Allyn, Buckinghamshire, UK) assessed at the renal transplant clinic.

A fasting blood sample was obtained from each participant and stored at -70°C until biochemical analysis.

### Biochemical Analyses

VCAM and ICAM were measured in plasma samples using a commercially available solid phase sandwich ELISA technique (Diaclone; available from IDS, Tyne and Wear, UK). The within run coefficient of variation for VCAM was 3% and the between run coefficient of variation was 10%. The within run coefficient of variation for ICAM was 1.2% and the between run coefficient of variation was 7.1%.

Serum total cholesterol and high density lipoprotein (HDL) cholesterol were measured using VITROS slides and analysed using a VITROS 700 System (Ortho Clinical Diagnostics, Rochester, NY, USA).

Serum creatinine was measured using the VITROS Slide System and the VITROS 950 analyser system (Ortho Clinical Diagnostics, Rochester, NY, USA). Detection range for creatinine was 4 - 1238 μmol/l and within lab coefficient of variation was 1.1%. Estimated glomerular filtration rate (eGFR) was calculated for all patients using the 4-variable MDRD equation [19]:

High sensitivity C reactive protein (hsCRP) was measured using a high sensitivity immunoturbidimetric assay (Randox, Crumlin, UK). Samples were analysed using a Roche Cobas Fara (Roche, Basel, Switzerland).

All 378 participants had measurements for VCAM, ICAM, total cholesterol, HDL cholesterol and creatinine. 375 participants had a measurement for hsCRP.

### Prospective Data Collection

The collection of prospective follow-up data was completed in April 2008 at a mean of 2243 days and a median of 2441 days after enrolment. The longest period of follow-up after recruitment to the study was 2844 days. Mortality data, including date of death, where applicable, was available for all participants. This information was obtained from the mortality data recorded on the Regional Nephrology Database at Belfast City Hospital and via letter and direct telephone contacts with the Primary Care Physicians of the renal transplant recipients enrolled in this study. No patients were lost to follow up.

### Statistical Analyses

Data analysis was performed using SPSS (version 11.0 Chicago, Illinois, USA). Kolmogorov-Smirnoff analysis was used to test if variables were normally distributed. Logarithmic transformation was performed for variables that did not conform to a normal distribution. For normally distributed variables data is expressed as arithmetic mean +/- standard deviation (SD). For those variables that were not normally distributed data is expressed as median with the interquartile range in brackets. The significance of differences between two groups was assessed using independent samples t-test for normally distributed variables. A two-tailed p value <0.05 was considered to be statistically significant.

Kaplan-Meier analysis with log rank test was used for univariate survival analysis. As there are no established reference values for either VCAM or ICAM, these variables were banded into thirds prior to inclusion in survival analysis.

A Cox Regression model was used for multivariate survival analysis. As cardiovascular disease is the leading cause of death in patients with a renal transplant [20], multivariate survival analysis was performed including traditional cardiovascular risk factors as co-variates. Given the size of the study population, traditional cardiovascular risk factors were banded into thirds prior to inclusion in the Cox Regression model. Since the risk of cardiovascular disease is also associated with hsCRP and eGFR, these variables were also included as co-variates, banded into thirds, in the Cox Regression analysis.

## Results

Characteristics of the participants at enrolment to this study are shown in Table 1.

**Table 1:** Biological and biochemical characteristics of the renal transplant recipients enrolled in this study

Characteristic	Distribution
Age (yrs)	47.3 +/- 14.3
Systolic blood pressure (mmHg)	132 +/- 14
Diastolic blood pressure (mmHg)	80 +/- 8
Total cholesterol (mmol/l)	5.2 +/- 1.0
HDL cholesterol (mmol/l)	1.4 +/- 0.4
Creatinine (μmol/l) eGFR (ml/min/m^2^)	130 (106-160) 52.5 +/- 20.2
hsCRP (mg/l)	1.9 (0.9, 4.6)
VCAM (ng/ml)	1510 (1151, 2156)
ICAM (ng/ml)	928 (664, 1263)
Time post transplant (years)	7 (3-13)
Time on dialysis pre-transplant (months)	13 (8-27)

Results expressed as arithmetic mean +/- standard deviation or median (inter-quartile range).

All participants were white, 243 (64%) were male, 72 (19%) were smokers and 54 (14%) had diabetes. 12 (3%) participants had received a pre-emptive renal transplant and 366 (97%) participants had been on dialysis prior to renal transplantation.

A minority, 84 (22%), of participants had a known history of cardiovascular disease at enrolment. Of those with a history of cardiovascular disease at enrolment, VCAM but not ICAM, was significantly higher as compared to those who had no known history of cardiovascular disease at enrolment (VCAM: 1731, (1290, 2666) ng/ml vs. 1443 (1127, 2097) ng/ml p = 0.011 and ICAM: 993 (740, 1366) ng/ml vs. 917 (635, 1256) ng/ml p = 0.151).

As participants were transplanted between 1968 and 2001, their immunosuppression mainly reflected the drug therapy available at the time of transplantation. Consequently wide combinations of immunosuppression regimens were in use in this study population. However, 258 renal transplant recipients were prescribed a calcineurin inhibitor. There was no significant difference in VCAM or ICAM concentration in those transplant recipients prescribed a calcineurin inhibitor as compared to those not prescribed a calcineruin inhibitor (VCAM: 1505 (1167, 2182) ng/ml vs. 1564 (1062, 2108) ng/ml p = 0.334 and ICAM: 909 (628, 1229) ng/ml vs. 976 (682, 1445) ng/ml p = 0.102).

At follow-up 305 participants were alive and 73 participants had died. Based on survival at follow-up patients were divided into two groups; group 1, deceased (n = 73) and group 2, survivors (n = 305). As shown in Table 2, significant differences included that those who had died during follow-up were older, more likely to be diabetic, have had a history of cardiovascular disease at enrolment, had lower eGFR, higher systolic blood pressure, and higher hsCRP levels as compared to those who were still alive at follow-up. Similarly, VCAM and ICAM concentration at enrolment were significantly higher in those renal transplant recipients who had died at follow-up. There was no significant difference in time from renal transplant in those participants who had died at follow-up as compared to those who were still alive at follow-up: 9 (4-14) years vs. 7 (3-12.5) respectively, p = 0.056,

**Table 2:** Differences in the renal transplant recipients who had died compared to those alive at follow-up

	Deceased (n = 73)	Survivors (n = 305)	Significance
Age (yrs)	57.4+/- 14.1	45.5 +/- 13.5	<0.001**
Male gender Cardiovascular disease at enrolment	45 33	198 51	0.773 <0.001**
Systolic blood pressure (mmHg)	135 +/- 14	131 +/- 14	0.031*
Diastolic blood pressure (mmHg)	78 +/- 6	79 +/- 7	0.067
Total cholesterol (mmol/l)	5.2 +/- 1.1	5.3 +/- 1.0	0.478
HDL cholesterol (mmol/l)	1.4 +/- 0.5	1.4 +/- 0.4	0.717
Diabetic	17	37	0.014*
Smokers	13	59	0.764
Creatinine (μmol/l) eGFR (ml/min/m^2^)	144(110-212) 45.9+/- 24.2	129(106-154) 53.9 +/- 19.0	0.011* 0.009**
hsCRP (mg/l)	3.8 (1.9, 9.9)	1.7 (0.8, 3.9)	<0.001**
VCAM (ng/ml)	2120(1476, 3136)	1428 (1109, 2025)	<0.001**
ICAM (ng/ml)	1061 (816, 1415)	900 (610, 1248)	0.003**

Results expressed as arithmetic mean +/- standard deviation or median (inter-quartile range). Levels of significance (* p < 0.05, ** p < 0.01).

Of the 73 renal transplant recipients who had died, 27 had died of a cardiovascular cause, 36 from a non-cardiovascular cause and for 10 participants cause of death could not be accurately established. There was no significant difference in VCAM or ICAM concentration in those renal transplant recipients who had died of a cardiovascular cause as compared to those who had died of a non-cardiovascular cause (VCAM 2055 (1422, 2759) ng/ml vs. 2478 (1340, 3329) ng/ml p = 0.54 and ICAM 985 (797, 1300) ng/ml vs. 1151(935, 1634) ng/ml p = 0.10).

**Figure 1:**
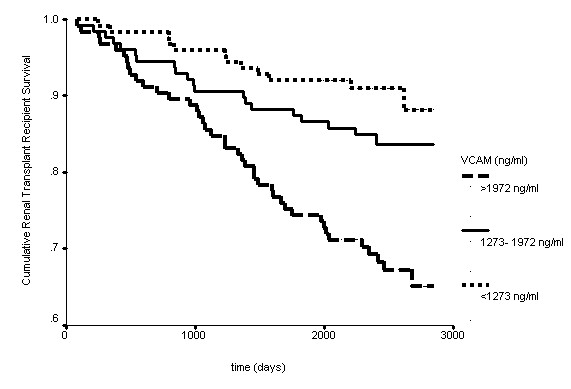
**Kaplan-Meier survival curve for renal transplant recipients stratified by VCAM concentration banded into thirds (p < 0.001)**.

**Figure 2:**
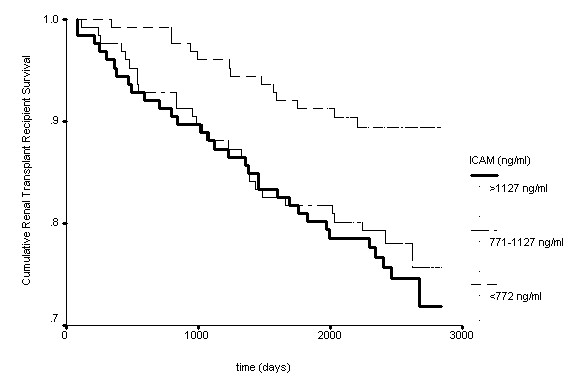
**Kaplan-Meier survival curve for renal transplant recipients stratified by ICAM concentration banded into thirds (p = 0.009)**.

As shown in Figure 1 and Figure 2, in univariate survival analysis those renal transplant recipients with a VCAM or ICAM concentration in the lowest third were significantly more likely to have survived at follow-up as compared to those transplant recipients with a VCAM or ICAM concentration in the middle or highest third.

In multivariate Cox Regression analyses, as shown in Table 3 and Table 4, VCAM and ICAM remained significant predictors of mortality following adjustment for traditional cardiovascular risk factors, hsCRP and eGFR.

**Table 3:** Renal transplant recipient survival analyses stratified by VCAM (abbreviation: CI, confidence interval)

	Kaplan-Meier	Cox Regression Analysis		
	
	Significance	Significance	Exp(B)	CI
VCAM^a^				
<1272 ng/ml	<0.001**	0.001**	1	
1272-1972 ng/ml			1.7	0.83, 3.51
>1972 ng/ml			3.3	1.7, 6.4

VCAM^b^				
<1272 ng/ml	<0.001**	0.030*	1	
1272-1972 ng/ml			1.5	0.73, 3.16
>1972 ng/ml			2.4	1.21, 4.81
VCAM^c^				
<1272 ng/ml	<0.001**	0.049*	1	
1272-1972 ng/ml			1.4	0.6, 2.9
>1972 ng/ml			2.2	1.1, 4.5

Levels of significance (* p < 0.05, ** p < 0.01).

^a^Cox Regression analysis adjusted for age, gender, smoking, diabetes, systolic blood pressure, total cholesterol and HDL cholesterol.

^b^Cox Regression analysis adjusted for age, gender, smoking, diabetes, systolic blood pressure, total cholesterol, HDL cholesterol, eGFR and hsCRP.

^c^Cox Regression analysis adjusted for age, gender, smoking, diabetes, systolic blood pressure, total cholesterol, HDL cholesterol, eGFR, hsCRP and history of vascular disease at enrolment

**Table 4:** Renal transplant recipient survival analyses stratified by ICAM (abbreviation: CI, confidence interval)

	Kaplan-Meier	Cox Regression Analysis		
	
	Significance	Significance	Exp(B)	CI
ICAM^a^				
<771 ng/ml	0.009**	0.021*	1	
771- 1127 ng/ml			2.39	1.22, 4.70
>1127 ng/ml			2.38	1.24, 4.59

ICAM^b^				
<771 ng/ml	0.009**	0.037*	1	
771- 1127 ng/ml			2.04	1.02, 4.06
>1127 ng/ml			2.37	1.22, 4.61
ICAM^c^				
<771 ng/ml	0.009**	0.021*	1	
771- 1127 ng/ml			2.39	1.22, 4.70
>1127 ng/ml			2.30	1.22, 4.57

Levels of significance (* p < 0.05, ** p < 0.01).

^a^Cox Regression analysis adjusted for age, gender, smoking, diabetes, systolic blood pressure, total cholesterol and HDL cholesterol.

^b^Cox Regression analysis adjusted for age, gender, smoking, diabetes, systolic blood pressure, total cholesterol, HDL cholesterol, eGFR and hsCRP.

^c^Cox Regression analysis adjusted for age, gender, smoking, diabetes, systolic blood pressure, total cholesterol, HDL cholesterol, eGFR, hsCRP and history of vascular disease at enrolment.

As VCAM was significantly higher in those renal transplant recipients who had a history of cardiovascular disease at enrolment, history of vascular disease at enrolment was also included as a co-variate in survival analysis. VCAM and ICAM remained significant predictors of outcome following adjustment for history of vascular disease at enrolment (Table 3 and Table 4).

## Discussion

Although cardiovascular disease remains the leading cause of death in patients with a renal transplant, traditional cardiovascular risk factors do not adequately account for the increased prevalence of vascular disease in this population [4]. An improved understanding of the pathophysiology of cardiovascular disease in this population is therefore critical to help identify mechanisms by which the burden of vascular disease could be reduced.

Although there have been prior publications reporting associations between CAMs and mortality in chronic renal failure and dialysis patients [1-3], the predictive value of CAMs has not been well defined in renal transplant recipients. We hypothesised that CAMs would predict risk of death in patients with a renal transplant and the aim of this study was therefore to assess the relationship between the CAMs, ICAM and VCAM, and all-cause mortality during the prospective follow-up of a renal transplant cohort.

CAMs are essential mediators in the process of atherosclerosis [6]. However, although elevated concentrations of CAMs are found in patients with atherosclerosis [21], in our study we found that VCAM, but not ICAM, was significantly higher in those renal transplant recipients with a history of cardiovascular disease at enrolment.

Nevertheless, in patients with chronic kidney disease, VCAM and ICAM have been shown to be independent predictors of mortality [2] and in the renal transplant recipients enrolled in our study VCAM and ICAM were indeed significant predictors of all-cause mortality.

Although it has been reported that the predictive power of CAMs for coronary artery disease is markedly attenuated following adjustment for traditional cardiovascular risk factors [22] VCAM and ICAM concentration remained significant predictors for mortality in the renal transplant recipients enrolled in our study even after adjustment for these risk factors.

Interestingly, although CAMs are associated with cardiovascular disease, there was no significant difference in VCAM or ICAM concentration in those renal transplant recipients who had died of a cardiovascular cause as compared to those who had died of a non-cardiovascular. However, as there were relatively few deaths in each of these 2 groups, we hypothesise that further follow-up may yield more significant results.

Elevated concentrations of CAMs are found in patients with chronic kidney disease [17,18,23]. However, in the renal transplant recipients enrolled in our study, VCAM and ICAM remained significant predictors of outcome following adjustment for renal function (glomerular filtration rate). Therefore, despite the association reported between renal function and CAM concentration, the data from our study demonstrate that CAMs provide additional prognostic information independent of renal function.

Elevated levels of ICAM and particularly VCAM have been found to be significantly related to future death from cardiovascular causes among patients with documented coronary artery disease [16]. In the renal transplant recipients enrolled in this study, those with a VCAM or ICAM concentrations in the lowest third had the best survival. Of note, risk of mortality showed a step-wise increase with increasing concentration of VCAM. However, the risk of all-cause mortality in renal transplant recipients with an ICAM concentration in the middle third was similar to the risk for those with an ICAM concentration in the highest third.

We believe that this two-centred prospective study, representative of the renal transplant recipients in Northern Ireland has yielded interesting results. Confounding factors were limited by recruiting patients with stable graft function who were clinically well, more than 2 months post-transplant, on standard immunosuppression regimens. In addition, the significant biological and biochemical differences between survivors and deceased were included as co-variates in multivariate Cox Regression survival analysis. Selection bias was minimised by lack of formal exclusion criteria.

We acknowledge that there are some limitations to our study. This study was performed in a white renal transplant population in one geographical region of the United Kingdom. Therefore the applicability or generalisability of the results of this study to other geographical regions or ethnic groups is not established. Also, although data on history of cardiovascular disease was recorded, information on other co-morbidities which could impact on mortality was not recorded at enrolment.

Nevertheless, this prospective cohort study of renal transplant recipients has generated interesting and significant results linking elevated concentrations of CAMs with subsequent mortality.

## Conclusion

CAMs are biochemical markers which reflect underlying endothelial activation and vascular inflammation [9]. Our study provides support for these as key mechanisms contributing to injury and death in renal transplant recipients. We believe we are the first group to show that the CAMs, ICAM and in particular VCAM, are significant independent predictors of all-cause mortality in patients with a renal transplant. We believe that the results of this study are worthy of further investigation in another population of renal transplant recipients and identifying agents which target these processes may prove to be an important strategy to improve the longevity of patients with a renal transplant.

## Abbreviations

**CAM**: cellular adhesion molecule; **CAMs**: cellular adhesion molecules; **CI**: Confidence Interval; **eGFR**: estimated glomerular filtration rate; **HDL**: high density lipoprotein; **hsCRP**: high sensitivity C reactive protein; **ICAM**: soluble intercellular CAM-1; **VCAM**: soluble vascular CAM-1.

## Competing interests

The authors declare that they have no competing interests.

## Authors' contributions

APM and ISY conceived and designed the study, contributed to the interpretation of data, revised the manuscript and provided intellectual content of critical importance to the work described. GMC contributed to the analysis and interpretation of data, drafted the article and provided intellectual content of critical importance to the work described. RC contributed to the study design, analysis and interpretation of the data, drafted the article and provided intellectual content of critical importance to the work described. PTM contributed to the analysis of data, revision of the manuscript and provided intellectual content of critical importance to the work described. All authors read and approved the final manuscript.

## Authors' information

GMC: Specialist Registrar in Clinical Biochemistry (Belfast Trust), MD, FRCPath, MRCP

RC: Consultant Nephrologist (Northern Trust), PhD, MRCP

PTM: Consultant Nephrologist (Belfast Trust), MD, MRCP

ISY: Professor of Clinical Biochemistry (Queen's University of Belfast), Consultant in Clinical Biochemistry (Belfast Trust), MD, FRCPath, FRCP

APM: Professor of Nephrology (Queen's University of Belfast), Consultant Nephrologist (Belfast Trust), PhD, MD, FRCP

## Pre-publication history

The pre-publication history for this paper can be accessed here:

http://www.biomedcentral.com/1471-2369/12/23/prepub

## References

[B1] Stenvinkel P Lindholm B Heimburger M Heimburger O Elevated serum levels of soluble adhesion molecules predict death in pre-dialysis patients: association with malnutrition, inflammation, and cardiovascular disease Nephrol Dial Transplant 2000 15 10 1624 1630 10.1093/ndt/15.10.1624 11007832

[B2] Suliman ME Qureshi AR Heimburger O Lindholm B Stenvinkel P Soluble adhesion molecules in end-stage renal disease: a predictor of outcome Nephrol Dial Transplant 2006 21 6 1603 1610 10.1093/ndt/gfl005 16476720

[B3] Papagianni A Dovas S Bantis C Belechri AM Kalovoulos M Dimitriadis C Efstratiadis G Alexopoulos E Memmos D Carotid atherosclerosis and endothelial cell adhesion molecules as predictors of long-term outcome in chronic hemodialysis patients Am J Nephrol 2008 28 2 265 274 10.1159/000110895 17989499

[B4] Foley RN Parfrey PS Sarnak MJ Clinical epidemiology of cardiovascular disease in chronic renal disease Am J Kidney Dis 1998 32 5 Suppl 3 S112 9 9820470 10.1053/ajkd.1998.v32.pm9820470

[B5] Libby P Ridker PM Maseri A Inflammation and atherosclerosis Circulation 2002 105 9 1135 1143 10.1161/hc0902.104353 11877368

[B6] Price DT Loscalzo J Cellular adhesion molecules and atherogenesis Am J Med 1999 107 1 85 97 10.1016/S0002-9343(99)00153-9 10403357

[B7] Blake GJ Ridker PM Novel clinical markers of vascular wall inflammation Circ Res 2001 89 9 763 771 10.1161/hh2101.099270 11679405

[B8] Gearing AJ Hemingway I Pigott R Hughes J Rees AJ Cashman SJ Soluble forms of vascular adhesion molecules, E-selectin, ICAM-1, and VCAM-1: pathological significance Ann N Y Acad Sci 1992 667 324 331 10.1111/j.1749-6632.1992.tb51633.x 1285023

[B9] Blake GJ Ridker PM Inflammatory bio-markers and cardiovascular risk prediction J Intern Med 2002 252 4 283 294 10.1046/j.1365-2796.2002.01019.x 12366601

[B10] Komatsu S Panes J Russell JM Anderson DC Muzykantov VR Miyasaka M Granger DN Effects of chronic arterial hypertension on constitutive and induced intercellular adhesion molecule-1 expression in vivo Hypertension 1997 29 2 683 689 9040457 10.1161/01.hyp.29.2.683

[B11] Markovic S Raab M Daxecker H Griesmacher A Karimi A Muller MM In vitro effects of cyclosporin A on the expression of adhesion molecules on human umbilical vein endothelial cells Clin Chim Acta 2002 316 1-2 25 31 10.1016/S0009-8981(01)00732-X 11750271

[B12] McMurray RW Adhesion molecules in autoimmune disease Semin Arthritis Rheum 1996 25 4 215 233 10.1016/S0049-0172(96)80034-5 8834012

[B13] Dedrick RL Bodary S Garovoy MR Adhesion molecules as therapeutic targets for autoimmune diseases and transplant rejection Expert Opin Biol Ther 2003 3 1 85 95 10.1517/14712598.3.1.85 12718733

[B14] Hauser IA Riess R Hausknecht B Thuringer H Sterzel RB Expression of cell adhesion molecules in primary renal disease and renal allograft rejection Nephrol Dial Transplant 1997 12 6 1122 1131 10.1093/ndt/12.6.1122 9198039

[B15] Bechtel U Scheuer R Landgraf R Konig A Feucht HE Assessment of soluble adhesion molecules (sICAM-1, sVCAM-1, sELAM-1) and complement cleavage products (sC4d, sC5b-9) in urine. Clinical monitoring of renal allograft recipients Transplantation 1994 58 8 905 911 10.1097/00007890-199410270-00008 7524208

[B16] Blankenberg S Rupprecht HJ Bickel C Peetz D Hafner G Tiret L Meyer J Circulating cell adhesion molecules and death in patients with coronary artery disease Circulation 2001 104 12 1336 1342 10.1161/hc3701.095949 11560847

[B17] Bonomini M Reale M Santarelli P Stuard S Settefrati N Albertazzi A Serum levels of soluble adhesion molecules in chronic renal failure and dialysis patients Nephron 1998 79 4 399 407 10.1159/000045084 9689154

[B18] Rabb H Calderon E Bittle PA Ramirez G Alterations in soluble intercellular adhesion molecule-1 and vascular cell adhesion molecule-1 in hemodialysis patients Am J Kidney Dis 1996 27 2 239 243 10.1016/S0272-6386(96)90547-8 8659500

[B19] Levey AS Bosch JP Lewis JB Greene T Rogers N Roth D A more accurate method to estimate glomerular filtration rate from serum creatinine: a new prediction equation. Modification of Diet in Renal Disease Study Group Ann Intern Med 1999 130 6 461 470 10075613 10.7326/0003-4819-130-6-199903160-00002

[B20] Briggs JD Causes of death after renal transplantation Nephrol Dial Transplant 2001 16 8 1545 1549 10.1093/ndt/16.8.1545 11477152

[B21] Rohde LE Lee RT Rivero J Jamacochian M Arroyo LH Briggs W Rifai N Libby P Creager MA Ridker PM Circulating cell adhesion molecules are correlated with ultrasound-based assessment of carotid atherosclerosis Arterioscler Thromb Vasc Biol 1998 18 11 1765 1770 9812916 10.1161/01.atv.18.11.1765

[B22] Malik I Danesh J Whincup P Bhatia V Papacosta O Walker M Lennon L Thomson A Haskard D Soluble adhesion molecules and prediction of coronary heart disease: a prospective study and meta-analysis Lancet 2001 358 9286 971 976 10.1016/S0140-6736(01)06104-9 11583751

[B23] Alcalde G Merino J Sanz S Zubimendi JA Ruiz JC Torrijos J de Francisco AL Cotorruelo JG Lopez-Hoyos M Novo MJ Circulating adhesion molecules during kidney allograft rejection Transplantation 1995 59 12 1695 1699 10.1097/00007890-199506270-00009 7541576

